# The scurs inheritance: new insights from the French Charolais breed

**DOI:** 10.1186/1471-2156-10-33

**Published:** 2009-07-06

**Authors:** Aurélien Capitan, Cécile Grohs, Mathieu Gautier, André Eggen

**Affiliations:** 1 INRA, UMR 1313 Génétique Animale et Biologie Intégrative, F-78350 Jouy-en-Josas, France; 2 AgroParisTech, UMR 1313 Génétique Animale et Biologie Intégrative, F-75231 Paris 05, France

## Abstract

**Background:**

Polled animals are valued in cattle industry because the absence of horns has a significant economic impact. However, some cattle are neither polled nor horned but have so-called scurs on their heads, which are corneous growths loosely attached to the skull. A better understanding of the genetic determinism of the scurs phenotype would help to fine map the polled locus. To date, only one study has attempted to map the *scurs *locus in cattle. Here, we have investigated the inheritance of the scurs phenotype in the French Charolais breed and examined whether the previously proposed localisation of the *scurs *locus on bovine chromosome 19 could be confirmed or not.

**Results:**

Our results indicate that the inheritance pattern of the scurs phenotype in the French Charolais breed is autosomal recessive with complete penetrance in both sexes, which is different from what is reported for other breeds. The frequency of the *scurs *allele (*Sc*) reaches 69.9% in the French Charolais population. Eleven microsatellite markers on bovine chromosome 19 were genotyped in 267 offspring (33 half-sib and full-sib families). Both non-parametric and parametric linkage analyses suggest that in the French Charolais population the *scurs *locus may not map to the previously identified region. A new analysis of an Angus-Hereford and Hereford-Hereford pedigree published in 1978 enabled us to calculate the frequency of the *Sc *allele in the Hereford breed (89.4%) and to study the penetrance of this allele in males heterozygous for both *polled *and *scurs *loci (40%). This led us to revise the inheritance pattern of the scurs phenotype proposed for the Hereford breed and to suggest that allele *Sc *is not fully but partially dominant in double heterozygous males while it is always recessive in females. Crossbreeding involving the Charolais breed and other breeds gave results similar to those reported in the Hereford breed.

**Conclusion:**

Our results suggest the existence of unknown genetics factors modifying the expression of the *scurs *locus in double heterozygous Hereford and Angus males. The specific inheritance pattern of the *scurs *locus in the French Charolais breed represents an opportunity to map this gene and to identify the molecular mechanisms regulating the growth of horns in cattle.

## Background

In cattle industry, absence of horns has a significant economic impact, since horns are a major cause of bruising and other injuries, which generate veterinarian costs and reduce the value of carcasses [1-3]. Furthermore, present feeding practices use head gates or retainers incompatible with horned animals. Finally, horned animals are potentially more dangerous to their handlers. Thus, removing horns of livestock not naturally polled, *i.e. *dehorning, is a general practice that remains a painful operation regardless of the method used [2,4,5]. It is usually perceived as only "treating the symptom and not the cause", since it must be repeated from generation to generation. Moreover, dehorning is one of the animal-welfare issues [3], and may be subject to reinforced legislation in the near future (*i.e*. within the European Community). Thus, breeding polled cattle offers an adequate non-invasive and long-term means to dehorning.

Inheritance of horns has been the subject of numerous studies and the most commonly accepted model has been proposed by White and Ibsen [6] and revised by Long and Gregory [7] and Brem *et al*. [8]. This model involves three loci, *polled*, *scurs *and *African horn*:

1. The *polled *locus has two alleles (Table 1): *P *(polled or absence of horns) dominant to *p *(horned).

**Table 1:** Horn and scurs inheritance models according to [7]^1 ^and [8]^2^

	*Sc/Sc*	*Sc/sc*	*sc/sc*
*P/P*	S	Male NS^1 ^or S^2^ Female NS	NS

*P/p*	S	Male S Female NS	NS

*p/p*	H	H	H

NS: non-scurred, S: scurred, H: horned.

2. The *scurs *locus has two alleles (Table 1): *Sc *coding for the development of scurs and *sc *for the absence of scurs. Scurs develop as small horn-like growths in the same area as horns but loosely attached to the skull [9-11]. They can vary in size and shape to look like horns and in some animals, partial fusion to the skull with age has been observed [12]. For the "scurs" phenotype to occur, the *polled *locus has to have at least one allele *P*. Allele *Sc *is dominant to allele *sc *in *P/p Sc/sc *males but recessive in *P/p Sc/sc *females. The scurs phenotype has been observed in numerous breeds such as Angus, Hereford, Fleckvieh, Simmental, Pinzgauer, Limousin, Charolais and some other cattle breeds.

3. The *African horn *locus has two alleles: *Ha *(presence of African horns) and *ha *(absence) with allele *Ha *dominant to allele *ha *in *P/p Ha/ha *males and recessive in *P/p Ha/ha *females.

In conclusion, based on different studies [6,7,13], it is generally believed that *scurs *and *African horn *loci are not alleles of the *polled *locus and do not modify the horn shape on an otherwise horned animal (*p/p*). Moreover, the fact that the expression of *scurs *and *African horn *loci is sex-influenced has led several authors to presume that they could be different alleles of the same locus [14]. Nevertheless, to our knowledge, no experimental design has ever permitted to resolve this question.

However, in different publications, exceptions to this model have been described [7,11,15,16]. Interestingly, Long and Gregory [7] have reported an exception to their rule, namely, a non-scurred *P/p Sc/sc *bull that produced scurred daughters. The authors conclude that this male was probably misclassified and thus did not pursue the study of this particular case further. Williams and Williams [11] have mentionned the existence of polled Hereford bulls producing only non-scurred offspring regardless of the type of cows they were bred to. One of these bulls, which had been intentionally mated to scurred dams and horned dams known to transmit the scurs phenotype, never produced scurred or horned bull-calves among its numerous offspring. This led the authors to suggest that the scurs allele is recessive to the absence of scurs instead of being dominant. They have also reported the following paradox: "*it is assumed that the scurless gene is sex-influenced. [Indeed additional] data show that there are more scurred males than females to substantiate this, but if this were so one would still expect to get scurred bulls from crosses which actually result in only polled offspring". *To explain this situation, the authors propose the putative existence of a new gene that would be epistatic to the *scurs *gene. Kräußlich and Röhrmoser [15] and Laminger [16] have also observed similar exceptions and have suggested that adding a maternal imprinting of the *Sc *allele in males to these models would solve the problem. In conclusion, all these inconsistencies put forward a lack of penetrance of the scurs phenotype in specific *Sc/sc *males.

Although the *polled *locus was one of the first mapped loci in cattle [14], to date and despite numerous studies over the past 15 years, the causal mutation has not been identified. One major problem is that scurs and horned phenotypes can be confused [12]. Clearly if the inheritance mode and the localisation of the *scurs *locus were better characterized, it would be easier to eradicate the scurs trait and to fine map the *polled *locus, especially in populations with a high frequency of scurred animals. However, only one study has reported the localization of the *scurs *locus on BTA19 (for *Bos taurus *chromosome 19) [1].

In this work, we had two objectives: (1) to study the inheritance of the scurs phenotype in the French Charolais breed using 297 offspring belonging to six paternal half-sib families, and (2) to validate or invalidate the localisation of the *scurs *locus on BTA19 (as described by Asai *et al*. [1]) by performing non-parametric and parametric linkage analyses on a sub-group of animals consisting of 267 offspring from 33 half-sib and full-sib families.

## Results and discussion

### Testing the inheritance patterns in French Charolais

The most commonly accepted model of the inheritance of *polled *and *scurs *loci is presented in Table 1: nine genotypes corresponding to the three phenotypes constitute the horn and scurs inheritance model originally proposed by White and Ibsen [6] based on Galloway-Holstein crosses and revised by Long and Gregory [7] based on Angus, Hereford and Angus-Hereford crosses and Brem *et al*. [8] based on observations in Fleckvieh.

Table 2 presents the phenotypes of the Charolais progeny born to polled-and-non-scurred (NS) bulls and horned *p/p *cows. Since these bulls produced offspring with horns (p/p) as well as polled (P/_) animals, they have to be considered as heterozygous for the *polled *locus (P/p). The results from Table 2 show three main discrepancies with the generally accepted inheritance pattern (Table 1):

**Table 2:** Results of the mating of non-scurred P/p French Charolais bulls (Figure 1) to horned cows

Bulls	Male progeny			Female progeny		
	
	NS	S	H	NS	S	H
*P/p Sc/sc*:						

5944	35	23	27	29	16	24

9952	1	0	2	1	2	1

16076	16	8	7	11	5	5

20433	7	2	2	10	4	1

20434	4	3	5	5	1	0

Total	63	36	43	56	28	31

*P/p sc/sc*:						

20444	14	0	5	13	0	8

Total	14	0	5	13	0	8

NS: non-scurred, S: scurred, H: horned.

**Figure 1:**
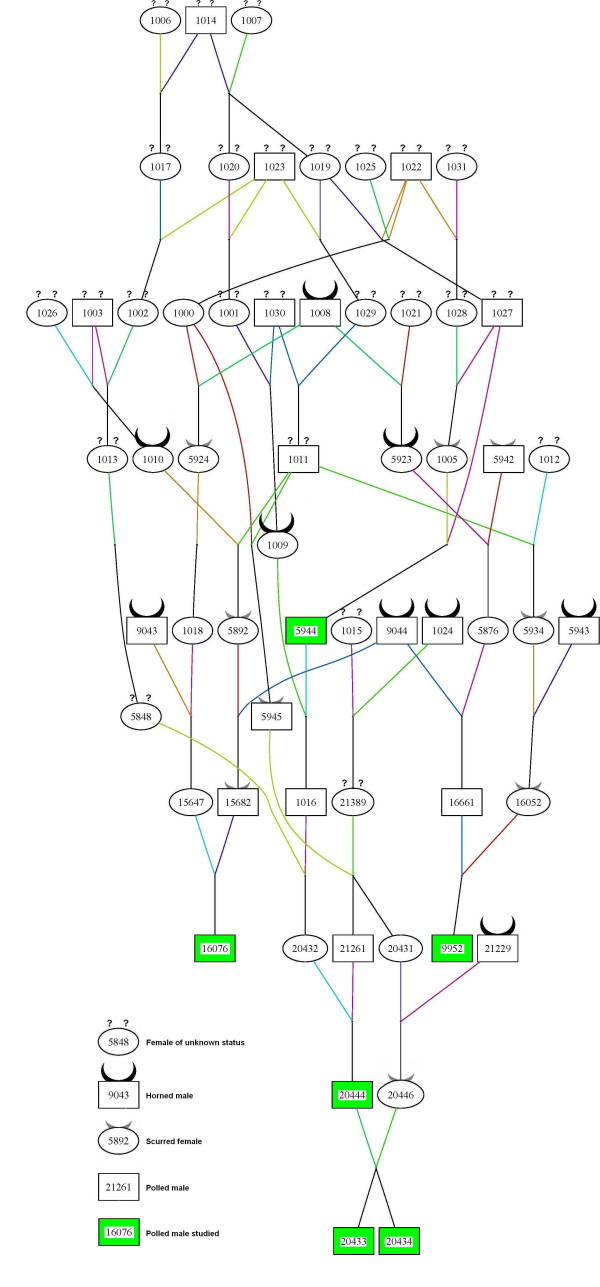
**Pedigree of the six sires from the FPCP nucleus**. The pedigree was designed using PEDIGRAPH 2.3 software [25]. Lines are drawn in different colours to better visualize the relationships between individuals.

i) According to this model, non-scurred *P/p *bulls are supposed to be *sc/sc *at the *scurs *locus. For this reason, they cannot have sired scurred *P/p *daughters since only *P/p Sc/Sc *females would be scurred. However, in our design this is the case for no less than five NS *P/p *bulls. Hence, these bulls must have one *Sc *allele, which they transmit to their progeny without expressing the scurs phenotype themselves. Among these bulls, sires 5944, 9952, 20433 and 20434 confirm this conclusion because born to a scurred mother (assumed to be *P/p Sc/Sc*), they have received at least one *Sc *allele (Figure 1). Thus, we have assumed that these five bulls are *P/p Sc/sc*.

ii) According to the model from Table 1, one should suppose that bull 20444 is *P/p sc/sc*; based on the high frequency of the *Sc *allele in the dam population, this bull should have sired at least some scurred *P/p Sc/sc *males among its 40 offspring, but none were observed. Actually, this bull did sire at least two polled non scurred but *P/p Sc/sc *bulls, namely, the above 20433 and 20434: both of them were born to a scurred mother and produced scurred male and female progeny when mated to the same type of dams as their father.

iii) Based on the same model, one expects more scurred males in the progeny of *P/p *sires because both *P/p Sc/sc *and *P/p Sc/Sc *males are supposed to express this phenotype whereas only *P/p Sc/Sc *females are supposed to do so. However, the progeny of the first five bulls analysed did not show any significant difference in the proportions of scurred sons (36/99 = 0.36) and daughters (28/84 = 0.33) among the non-horned progenies (chi square = 0.18, p > 0.05).

These three arguments led us to conclude that in the French Charolais breed, *P/p Sc/sc *males are in fact polled and not scurred. Moreover, since non-scurred *P/p *bulls mated to *p/p *cows can generate both scurred and non-scurred *P/p *progeny, the *scurs *locus cannot be an allele of the *polled *locus. Therefore, in this breed, the inheritance of the scurred condition is autosomal, and allele *sc *(absence of scurs) is completely dominant over allele *Sc *in both sexes (Table 3).

**Table 3:** Horn and scurs inheritance models according to the observations made in the French Charolais breed

	*Sc/Sc*	*Sc/sc*	*sc/sc*
*P/P*	S	NS	NS

*P/p*	S	NS	NS

*p/p*	H	H	H

NS: non-scurred, S: scurred, H: horned.

### Estimation of the *Sc *allele frequency in the French Charolais female population

The new simplified model allows us to estimate the frequency of the *Sc *allele in the Charolais female population, from the non-horned progeny of the five *P/p Sc/sc *bulls (Table 2). Based on our observations and assuming that the *scurs *locus is in Hardy-Weinberg equilibrium in the dam population, the frequency of the *Sc *allele is equal to 2*nS/(nS + nNS), where nS and nNS being respectively the number of scurred and non-scurred progeny of both sexes. Based on the total numbers given in Table 2, we can estimate the allelic frequency of *Sc *in the horned Charolais population: (2*(36+28)/(36+28+63+56)) *i.e. *69.9%.

### Revision of the previously proposed inheritance of the scurs phenotype

Since our results suggest that the inheritance of the scurs phenotype in the French Charolais breed may differ from that in other breeds, we decided to revise the data set studied by Long and Gregory [7] in the light of our results. Indeed, this data set constitutes a unique example of a large crossbreeding experimentation between polled and horned breeds focusing on the inheritance of both polled and scurs phenotypes. To verify if important deviations to the Long and Gregory model (as reported in section "Background") existed in their own design, we calculated the frequency of the *Sc *allele in the female Hereford population before confronting the real and the expected frequencies of scurred individuals among the male progeny.

### Estimation of the *Sc *allele frequency in horned Hereford

To estimate the frequency of the *Sc *allele in horned Hereford populations, we considered all the non-horned females sired by the nine *P/P Sc/sc *Angus bulls and their 11 Hereford counterparts (Table 4). To our knowledge, all suggested models for the inheritance of the scurs phenotype agree that *P/p *females must be *Sc/Sc *to express scurs.

**Table 4:** Results of the cross between P/P Angus and Hereford sires and horned Hereford dams^1^

Non-scurred sires	Male progeny			Female progeny		
	
	NS	S	H	NS	S	H
*P/P Sc/sc*:						

9 Angus	6	9	0	11	10	0

11 Hereford	9	10	0	15	11	0

Total	15	19	0	26	21	0

*P/P sc/sc*:						

42 Angus	48	29	1	72	0	0

18 Hereford	24	11	0	24	0	0

Total	72	40	1	96	0	0

^1 ^according to [7]. NS: non-scurred, S: scurred and H: horned.

Individuals scurred on one side were considered as scurred.

Animals registered by Long and Gregory [7] as scurred on only one "horn" were assumed to be *Sc/Sc *in all the following calculations since they are also *P/p*. Finally, we assumed that the determinism of the scurs phenotype is similar in Angus and Hereford breeds and that matings were randomly planned to permit an unbiased calculation of the *Sc *allele frequency.

On this basis, the frequency of the *Sc *allele in the horned Hereford female population equals 89.4% (*i.e. *2*nS/(nS + nNS) = 2*21/47). This high frequency explains the difficulties encountered during many decades in efforts to establish true polled Hereford strains from scurred individuals.

### Comparison between the expected and real frequencies of scurred male progeny

Considering that the frequency of the *Sc *allele is 89.4% in the horned female Hereford population, one expects a percentage of 89.4% of *P/p Sc/sc *individuals in the progeny of these dams mated to *P/P sc/sc *bulls. Thus, assuming a complete penetrance of the *Sc *allele in *P/p Sc/sc *males, one would expect the same frequency of scurred individuals among the bull-calves. However, according to the data reported by Long and Gregory [7], the observed frequency reaches 35.7% (40 scurred/112 scurred and non-scurred). This frequency is significantly different from the expected frequency (chi-square = 339, p < 10^-10^). Since Long and Gregory [7] have classified the bulls according to the mating results, it is highly improbable that this significant deviation is due to a difference between pools of mothers crossed with *P/P Sc/sc *and those crossed with *P/P sc/sc *bulls, or to a difference between pools of bull-calves or heifer's mothers because the gender of the progeny was randomly determined. Thus, we postulate that this deviation is most probably due to incomplete penetrance of the *Sc *allele at the heterozygous state in *P/p *males, and we estimate that only 40.0% (35.7%/89.4%) of the *P/p Sc/sc *males do express scurs in this data set.

Similar observations were made when analysing the progeny of *P/P Sc/sc *Angus and Hereford sires bred with Hereford dams or Angus-Hereford and Hereford-Hereford crosses independently (data not shown).

This enabled us to revisit the inheritance patterns of the scurs phenotype proposed by Long and Gregory [7] and Brem *et al*. [8], stating that *Sc *is not fully but partially dominant to sc in *P/p Sc/sc *males since both scurred and non-scurred *P/p Sc/sc *males exist.

### Linkage Analyses

Table 5 presents the genetic map built considering all 33 families simultaneously. The locus order is consistent with already published maps for this chromosome, *i.e.*, a map based on the physical distances using the bovine sequence assembly Btau_4.0, the USDA-MARC bovine genetic map [17,18] and the map built by Asai *et al*. [1]. However, due to a lack of recombinant individuals, some of the markers were completely linked in the present study and thus their order used in subsequent analyses was based on physical distances in the latest available genome assembly, Btau_4.0.

**Table 5:** Comparison of the genetic map built in the present study and published maps

	Genetic distances between markers (cM)			
	
Markers	Present study (informative meiosis)	Btau 4.0 assembly1	USDA-MARC genetic map	Asai *et al*., 2004
BMS1920^2^	0.0 (294)	0	0	0

DIK4306	0.0 (150)	1.2	3.0	/

CSSME070^2^	3.4 (179)	3.1	3.7	3.5

INRABTA19_01	3.4 (250)	4.5	/	/

BMS2142^2^	3.9 (209)	5.6	3.7	9.2

INRABTA19_02	3.9 (190)	5.8	/	/

INRABTA19_03	5.3 (272)	6	/	/

BP20^2^	7.6 (26)	6.2	6.3	17.7

BMS2503	9.3 (192)	8.9	11.8	/

BMS2389^2^	10.7 (354)	9.7	12.6	25.6

DIK5224	14.9 (134)	14.8	18.5	/

^1 ^assuming 1 Mb equivalent to 1 cM.

^2 ^markers also genotyped by [1].

**Figure 2:**
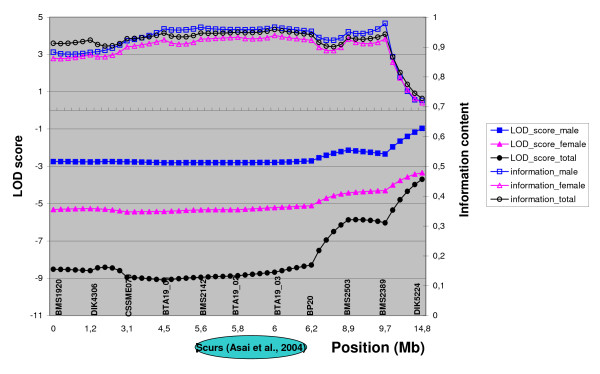
**Parametric linkage analyses of the 14.8-cM interval studied on BTA19**. The upper three curves represent the information content for males, females and all progeny combined (Y-axis on the right); the lower ones represent the LOD score as calculated by GENEHUNTER for males, females and all progeny combined (Y-axis on the left). Also presented is the putative *scurs *locus according to Asai et al., 2004.

Three different parametric linkage analyses were performed, on males and females separately and on both sexes combined, setting the frequency of allele *Sc *to 70% and considering allele Sc recessive to sc according to our observations. Penetrance values of 0.05, 0.05 and 0.9 were applied to *sc/sc*, *Sc/sc *and *Sc/Sc *genotypes, respectively (instead of 0, 0 and 1 as expected in the case of a recessive genetic determinism) to allow for some flexibility that may be caused by putative phenotyping errors. As shown in Figure 2, no significant linkage was found between the *scurs *locus and the region under investigation. Moreover, the maximum LOD score values reached high negative values (-2.71, -5.11 and -8.28, respectively for males, females and both), at the putative *scurs *locus (between 5.6 and 6.2 cM) of Asai *et al*. (2004).

**Figure 3:**
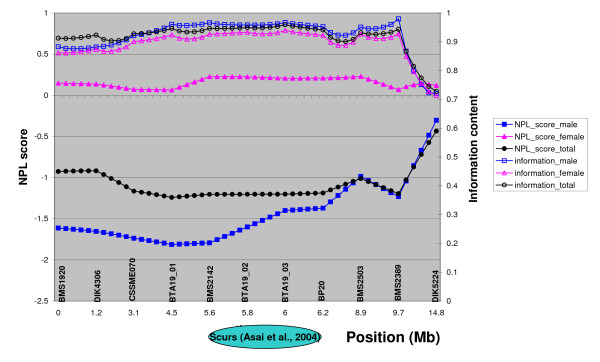
**Non-parametric linkage analyses of the 14.8 -cM interval of BTA19 studied**. The upper three curves represent the information content for males, females and all progeny combined (Y-axis on the right) and the lower ones the non-parametric scores as calculated by GENEHUNTER for females, males and all progeny combined (Y-axis on the left). Also presented is the putative *scurs *locus according to Asai et al., 2004.

The non-parametric linkage analyses performed on males, females and all progeny (Figure 3) fully agreed with these results, which enabled us to reject this region of BTA19 as a putative localisation for the *scurs *locus in our pedigree.

### Considerations on the localisation of the *scurs *locus by Asai *et al*., 2004

In their study, Asai *et al.*, 2004 [1] have analysed their first three crossbred families under the assumption of a sex-influenced inheritance of scurs via the dams. However, no specific detail is mentioned in the paper concerning the penetrance in their pedigree. Most probably, the genotypes of the male offspring at the *scurs *locus were deduced according to the inheritance model presented in Table 1. It is worth noting that: i) four of the six families they mentioned are from breeds in which inconsistencies to Long and Gregory and Brem *et al. *models have already been reported (*i.e. *Simmental [15,16] and Angus [19]) and ii) their nine non-scurred and twenty one scurred bull-calves are all *P/p *at the polled locus and thus potentially subject to incomplete penetrance. This may explain why we were not able to confirm the localisation of the *scurs *locus on BTA19 with our full French Charolais pedigree. Another explanation could be the existence of a different locus in French Charolais cattle. However, this seems very unlikely as discussed in the following section.

### Proposed hypotheses to explain the determinism of the scurs phenotype

Based on the results from previous studies together with our observations, we have made the following additional hypotheses in order to explain differences observed between the inheritance pattern of the scurs trait in French Charolais and other breeds:

(i) a new *scurs *locus or (ii) a new allele at the *scurs *locus may be responsible for the specific inheritance of the scurs phenotype in the Charolais breed;

iii) *scurs *and *polled *loci in the French polled Charolais may interact differently;

iv) a new biallelic locus may be responsible for the sex-influenced expression of the *Sc *allele in *P/p Sc/sc *males in the Angus and Hereford populations; this locus would be fixed in the French Charolais breed;

v) another locus located on chromosome X with two alleles may be involved: *X*_*Sc *_would be responsible for the expression of *Sc *in *P/p Sc/sc *individuals of both sexes and *X*_*sc *_would be fixed in the French Charolais breed, whereas both alleles would segregate in Angus, Hereford and other breeds;

vi) due to maternal imprinting, *P/p Sc/sc *males may express the scurs phenotype only if they receive the *Sc *allele from their mother whereas all *P/p Sc/sc *females do not express it;

vii) due to paternal imprinting, the *P/p Sc/sc *males may express the scurs phenotype only if they receive the *Sc *allele from their father whereas all *P/p Sc/sc *females do not express it.

Some of these hypotheses could be tested by studying the offspring of Charolais animals bred to animals from other breeds (Table 6).

**Table 6:** Results from different Charolais crosses

	Male progeny			Female progeny		
	
Crosses	NS	S	H	NS	S	H
1 *P/p Sc/Sc *Charolais bull^1 ^× 6 *p/p sc/sc *Holstein cows^2^	1	2	1	1	0	1

1 *P/p Sc/Sc *Charolais bull^1 ^× 5 *p/p -/- *Montbéliard cows^3^	0	1	2	2	0	0

**Total**	**1**	**3**	**3**	**3**	**0**	**1**

1 *P/P sc/sc *Angus bull × 1 *p/p Sc/- *Charolais cow^4^	5	5	0	7	0	0

**Total**	**5**	**5**	**0**	**7**	**0**	**0**

NS: non-scurred, S: scurred and H: horned.

^1 ^This scurred bull (#5945 in **Figure 1**) was assumed to be *P/p Sc/Sc *according to the inheritance pattern for the *scurs *locus in the French Charolais breed.

^2 ^In modern Holstein populations, allele *Sc *appears to be rare [20].

^3 ^Data on the frequency of the scurs phenotype in this horned breed are not available.

^4 ^Family CRBH3 [1,21]; assuming the specific inheritance of the scurs trait in the French Charolais breed, probabilities that this Charolais dam is *p/p Sc/sc *as assumed by Asai *et al. *[1] or *p/p Sc/Sc *are equal.

1. Observations in Charolais × Holstein and Angus × Charolais crosses [1,20,21] suggest that in crossbred progeny, the inheritance of the Charolais scurs trait is more likely in agreement with the pattern encountered in other breeds (*i.e. *recessivity of the Sc allele in females and dominance in males rather than recessivity in both sexes as observed in French Charolais) and that it is not dependent on the origin of the P allele. Such observations lead us to reject hypotheses i, ii, and iii.

2. In the Angus × Charolais cross, all the P/p Sc/sc males have received a *X*_*sc *_from the Charolais dam and thus are supposed not to express the scurs trait under hypothesis v). The existence of five scurred males born from this mating invalidates this hypothesis.

3. The Charolais × Hosltein crossbreeding experiment clearly shows that two scurred P/p Sc/sc males have received allele Sc from their father [20], thus hypothesis vi *i.e. *inheritance through the dam (vi) is rejected.

4. Since the scurred males born from the Angus × Charolais mating did not receive any allele Sc from their father, hypothesis vii) is false.

Given the assumptions we made on the founder genotypes, only hypothesis iv) is never rejected. However, it must be noted that the progeny number of Charolais crossbred is currently too low to draw any definite conclusion. It would be of great interest to increase this number to better understand the mechanisms responsible for the sex-influenced expression of the scurs locus in certain P/p Sc/sc males.

Moreover, to date, it has not been possible to explore all the genetic combinations in a single study to definitely prove the accuracy of the successive models proposed. Even the combination of all published data does not provide a clear picture on the genetic determinism responsible for the scurs phenotype. The structure of commercial cattle populations presents several constraints. For example, it is almost impossible to study the maternal transmission of the scurs phenotype because of the low number of progeny available per dam. A better understanding of this maternal transmission and the localisation of the locus would undoubtedly help to define the specific determinism of the scurs phenotype as in the case of the callipyge phenotype in sheep [22].

## Conclusion

This article examines the inheritance pattern of the scurs phenotype in French Charolais cattle and concludes that in this population it is autosomal recessive with complete penetrance in both sexes. Our results differ slightly from the inheritance pattern proposed for other populations by Long and Gregory [7] and Brem *et al*. [8], presenting the *Sc *allele as dominant in males and recessive in females in double heterozygous animals for the *polled *and *scurs *loci. A partial review and analysis performed on their data set enabled us to revise that model, suggesting that the *Sc *allele is most probably dominant in specific males and recessive in other males. Different crosses involving Charolais cattle have given similar results. We have demonstrated that the difference between the inheritance pattern in the Charolais breed and in other breeds is not due to particular Charolais *scurs *or *polled *alleles/loci. To date, the reason why this difference exists remains unknown. Finally, the specific inheritance pattern of the scurs phenotype in the French Charolais breed offers a promising approach to fine map the *scurs *locus and to identify the molecular mechanism regulating the growth of horns in cattle.

## Methods

### Pedigree material and phenotypes

The present study was conducted in collaboration with a French Polled Charolais Program (FPCP). This program, initiated in 1993, aims at introgressing the polled condition in a commercial population. For this purpose, in the first generation, high genetic value sires were mated to polled and scurred Charolais cows. In the next generation, heterozygous polled animals were crossbred to maximize the generation of polled males that could become progeny tested bulls of high genetic value (carcass and maternal traits) for future artificial insemination (AI). Many animals have already been genotyped for microsatellites from the polled region located in the centromeric region of BTA01 [23,24].

Experiments reported in this work comply with the French National Institute for Agricultural Research (INRA) ethical guidelines.

### Phenotypes

Because the growth of scurs occurs later in life than horns, animals were phenotyped twice: between four and six months and between nine and eighteen months. All types of corneous growths that were loosely attached to the skull were considered as scurs [9-11]. To avoid any confusion a non-scurred (NS) phenotype refers to the phenotype characterised by the absence of both horns and scurs, while the polled phenotype corresponds to the absence of true horns.

### Pedigree

The inheritance of the scurs phenotype was studied on 297 Charolais offspring belonging to six paternal half-sib families (ranging from seven to 154 offspring). These animals result from a cross between non-scurred *P/p *AI sires from the FPCP nucleus and 265 horned *p/p *dams originating from 153 different herds. These dams were chosen to be representative of the breed. However, this panel is characterized by an excess of polled and scurred versus horned offspring (70.7%), a bias that can be attributed to selection prior to sampling. Indeed, during the first wave of introgression, breeders kept all the polled and scurred animals of both sexes while they sold a number of horned calves. The parental origin of the sires of the half-sib families is presented in Figure 1. This pedigree was designed using PEDIGRAPH 2.3 software [25].

For linkage analysis, 267 animals (52 originating from the panel cited above and 215 from the FPCP nucleus breeding scheme) and most of their parents were genotyped. Among these 267 animals, 170 scurred and 97 non-scurred, all offspring can be classified in 33 half-sib and full-sib families.

Finally, for crossbreeding studies we considered 11 offspring from the mating of a scurred Charolais bull with respectively five and six unrelated horned Montbéliard and Holstein cows. This bull was considered to be genotypically *P/p Sc/Sc *according to observations in the French Charolais breed.

### DNA extraction, markers and genotyping

DNA samples were extracted from sperm using a standard phenol-chloroform method, or from blood using a non-organic-based extraction method [26].

Eleven microsatellites located on BTA19 were genotyped in this study. BMS1920, CSSME070, BMS2142, IDVGA46, BP20 and BMS2389 were chosen based on their chromosomal localization in a region 25.6 cM long and containing the *scurs *locus as previously described by Asai *et al*., 2004. Additional markers were selected based on their location on the USDA-MARC bovine genetic map [17,18] (DIK4306, BMS2503 and DIK5224) or identified from the bovine genome sequence assembly Btau_4.0 using tandem repeat finder version 4 [27] (INRABTA19_01, INRABTA19_02 and INRABTA19_03 [Genbank: FJ514780 to FJ514782]). IDVGA46 was discarded since it could not be amplified in our PCR conditions. PCR primers were designed with Primer 3 [28] and their sequences are shown in Table 7.

**Table 7:** Characteristics of the microsatellite markers

Markers	Forward primers 3'5' sequence	Reverse primers 3'5' sequence	Allele size range and (number)^1^
BMS1920	TCCCACCTACTTGGAAAATTG	ATGACTCAATGACCAACTGACC	116–126 (6)

DIK4306	ATGGTGGCAATGGAGATGAT	CATTCTTTCAGCTGCTAGGC	198–216 (6)

CSSME070	ATACAGATTAAATACCCACCTG	TTCTAACAGCTGTCACTCAGGC	139–145 (4)

INRABTA19_01	TTGAAGTTTCTGGGCTTAAGGA	TGTAGTTCTCAGGGCCAAGC	145–173 (11)

BMS2142	AAGCAGGTTGATGATCTTACCC	GTCGGCACTGAAAATGATTATG	85–115 (11)

INRABTA19_02	GACAAGAGGCTCTGAAGAGAGG	CATGTGTATTGGGTCTACAGCA	146–160 (6)

INRABTA19_03	TGAGACATGCATTCCCAAAT	GGCCTCCAGAACTGAGAGAA	195–245 (17)

BP20	TCTGTGGGTGAACAAGCAAG	GGCTCCCTAAAGACCCACTC	228–242 (6)

BMS2503	TTGAACAACTACCAGCTTCCC	GACATGACTGAGCGCGTG	162–172 (5)

BMS2389	AATGTTAGGTTTACATGCAGCC	AGGCAATAGGATCTCCACTAGC	91–113 (11)

DIK5224	TCTAGCTTCTCGGAGGTTGC	CCTGGAATAGGTGGACCCTTA	167–197 (7)

^1 ^according to genotyping results in our population.

The genotyping procedure consisted of a multiplex fluorescent PCR amplification (primers from Eurogentec, Angers, France), using the Multiplex PCR kit (QIAGEN, Hilden, Germany) according to the manufacturer's recommendations, on a PTC-100 thermocycler (MJ Research, Paris, France). The resulting PCR products were purified on Sephadex G50 columns (Amersham, Paris, France) before running on a MegaBACE 96 capillaries sequencer (Molecular Dynamics, Paris, France). Raw data were then analyzed using Genetic Profiler v1.5 (Molecular Dynamics).

### Linkage analyses

For linkage analysis, we used a map based on the bovine sequence assembly Btau_4.0, assuming 1 Mb equivalent to 1 cM. Marker order and map distances were validated using CRIMAP 2.4 software [29]. The FLIPS option with a five-marker window was used to obtain the most likely order given our data set. The CHROMPIC option helped identification of unlikely double crossovers.

Non-parametric linkage (NPL) analysis and parametric linkage analysis (logarithm of odds score analysis) were performed using GENEHUNTER software [30]. In cases where families were too large, they were split into separate subfamilies before analysis.

## Authors' contributions

AC participated in the design of the study and in data acquisition, performed animal genotyping and statistical analysis, interpreted the results and drafted the manuscript. CG extracted the DNA samples, participated in data acquisition and animal genotyping and revised the manuscript. MG participated in statistical analysis and revised the manuscript. AE conceived and coordinated the study and revised the manuscript. All authors read and approved the final manuscript.
